# Mammary Cells with Active Wnt Signaling Resist ErbB2-Induced Tumorigenesis

**DOI:** 10.1371/journal.pone.0078720

**Published:** 2013-11-12

**Authors:** Wen Bu, Xiang Zhang, Hua Dai, Shixia Huang, Yi Li

**Affiliations:** 1 Lester & Sue Smith Breast Center, Baylor College of Medicine, Houston, Texas, United States of America; 2 Department of Molecular and Cellular Biology, Baylor College of Medicine, Houston, Texas, United States of America; 3 Department of Physiology, School of Medicine, Yangzhou University, Yangzhou, Jiangsu, China; University of Texas Health Science Center, United States of America

## Abstract

Aberrant activation of Wnt signaling is frequent in human malignancies. In normal epithelial tissues, including the breast, Wnt signaling is active only in a subset of cells, but it is unknown whether this subset of Wnt signaling-active cells is at increased risk of carcinogenesis. We created transgenic mice (TOP-*tva*) in which the synthetic Wnt-responsive promoter TOP controlled the gene encoding TVA, which confers susceptibility to infection by the retroviral vector RCAS. Thus, only cells in which Wnt signaling is active will express *tva* and be targeted by RCAS. Surprisingly, we found that RCAS-mediated delivery of cDNA encoding a constitutively activated version of ErbB2 (HER2/Neu) into the small number of TVA+ mammary epithelial cells in TOP-*tva* mice failed to induce tumor, while the same virus readily induced mammary tumors after it was delivered into a comparable number of cells in our previously reported mouse line MMTV-*tva,* whose *tva* is broadly expressed in mammary epithelium. Furthermore, we could not even detect any early lesions or infected cells in TOP-*tva* mice at the time of necropsy. Therefore, we conclude that the Wnt pathway-active cell subset in the normal mammary epithelium does not evolve into tumors following ErbB2 activation–rather, they apparently die due to apoptosis, an anticancer “barrier” that we have reported to be erected in some mammary cells followed ErbB2 activation. In accord with these mouse model data, we found that unlike the basal subtype, ErbB2+ human breast cancers rarely involve aberrant activation of Wnt signaling. This is the first report of a defined sub-population of mammalian cells that is “protected” from tumorigenesis by a potent oncogene, and provides direct in vivo evidence that mammary epithelial cells are not equal in their response to oncogene-initiated transformation.

## Introduction

Members of the Wnt family are locally acting, extracellular matrix-binding glycoproteins that exert their biological effects by binding to their membrane receptors, the frizzled and low-density-lipoprotein receptor-related proteins (LRP5/6) [1]. As a result, β-catenin is stabilized, translocates to the nucleus, forms heterodimers with members of the TCF/LEF family of DNA-binding proteins, binds to the TCF binding motif in Wnt-responsive genes, and transactivates them [2]. Wnt signaling is important in many developmental processes including embryogenesis, hair follicle regeneration, colorectal epithelium renewal, and mammary gland formation [1], [3], [4]. It is normally active in a subset of cells in a given tissue type. Mutational and epigenetic events activating Wnt signaling are frequent in many human malignancies [1]. For example, Wnt signaling activation is detected in a subset of human breast cancer, most notably the basal subtype [5]–[10], although mutations of genes encoding Wnt signaling components are rare in human breast tumors [11], [12]. Numerous in vitro and in vivo experiments have demonstrated that aberrant activation of Wnt signaling causes or promotes cancer formation [2], [13]. More recent studies show that Wnt signaling activation is important in generating and maintaining the cancer stem cell population within a cancer [14]–[17].

Because Wnt signaling has a crucial role in carcinogenesis, the subset of cells with active Wnt signaling in a tissue may be at higher risk of cancer development than other cells with low or no Wnt signaling. This appears to be true in the intestine: intestinal cells that are positive for LGR5, a transcriptional target of Wnt signaling, are more easily induced to form cancer by ablation of *APC* than other cells in the same tissue [18]. Wnt signaling is active in a subset of cells in the mammary epithelium [19]–[24]. In this report, we tested whether Wnt signaling-active mammary epithelial cells are more or less susceptible to tumor induction by aberrant ErbB2 signaling than other cells in the mammary epithelium.

## Materials and Methods

### Ethics Statement

All procedures using mice were performed in compliance with a Baylor College of Medicine Animal Care and Use Committee-approved animal protocol (protocol number: AN-2834).

### Transgenic Mice and Animal Care

To create the TOP-*tva* transgenic construct, a PCR fragment from TOPdGFP [25] was first cloned into PCR2.1 vector using two primers, CAATTAACCCTCACTAAAGG and TCTTCGCTATTAC GCCAGTC. The DNA fragment containing the SV40 terminator and the TOP promoter containing 3 TCF binding sites and a *c-Fos* basic promoter were isolated from PCR2.1-TOP-*d2GFP* by Spe I and Xma I restriction enzymes, and then inserted in the MMTV-*tva* construct digested with Spe I and Xma I. From the resulting plasmid DNA, the vector DNA was removed by digestion with Bgl II. The remaining 2.1-kb DNA fragment contains the SV40 insulator, 3 TCF binding sites, *c-Fos* basic promoter, the *tva* cDNA, and the mouse protamine-1 poly (A) signal. This transgenic construct (TOP-*tva*) was injected into pronuclei from FVB/N mice. Potential founder mice were screened by PCR on tail DNA using oligos specific for the TOP-*tva* construct. MMTV-*Wnt1* transgenic mice have been reported; the line used here was on the FVB background and was purchased from Charles Rivers. MMTV-*tva* mice have been previously reported [26]. All mice were kept on 2920X Teklad Global Extruded Rodent Diet (Soy Protein-Free) (Harlan Laboratories, Indianapolis, IN).

### Generation of Single Mammary Gland Suspension Cells and Flow Cytometry

Generation of single mammary gland suspension cells has been reported previously [26]. The fluorescence-activated cell analysis was carried out using a BD LSRII (BD Bioscience, San Jose, CA). FACS Diva V6.1.2 software (BD Bioscience) was used for data analysis.

### Virus Preparation and Intra-ductal Infection of Mammary Glands

RCAS-*PyMT* has been described [27]. RCAS-*GFP* was a gift of Dr. Connie Cepko (Harvard Medical School, Boston, MA). Virus preparation has been previous described [28]. Virus titers were determined by limiting dilution on DF1 cells. To infect mammary glands, female mice were anesthetized and injected through intraductal injection [27], [28] with concentrated RCAS viruses in a 10-µl volume in conjunction with a tracking dye (0.1% bromophenol blue).

### Tissue Processing and Immunocytochemistry

Tissues were fixed and processed as described [26]. Immunohistochemistry and immunofluorescence were performed as described [26]. The following antibodies were used: purified rabbit antibodies against mouse keratin 6 (Covance, Princeton, NJ), keratin 5 (Covance), and TVA (a gift of Andy Leavitt, University of California, San Francisco); purified mouse monoclonal antibodies against α-smooth muscle actin (SMA, Dako, Carpinteria, CA); and partially purified rat antibodies against keratin 8, purchased from the Developmental Studies Hybridoma Bank, University of Iowa.

### Bioinformatic Analysis

The Concept Association Analysis was done through Oncomine (https://www.oncomine.com). The upregulated genes (more than 1.8 fold upregulated) of the MMTV-*Wnt1* transgenic mouse mammary glands vs. wild type mammary glands have been reported before [29]. These upregulated genes were uploaded as a concept of Wnt pathway-activated genes into the Oncomine. The significantly associated concepts were searched from the breast cancer datasets collected in the Oncomine.

We performed Gene Set Enrichment Analysis (GSEA) to test the enrichment of WNT activated genes in triple negative tumors in two datasets: ESK-MSK [30] and TCGA (cancergenome.nih.gov). ER and ERBB2 statuses were determined either by pathological annotation (EMC-MSK) or by expression values of *ESR1* and *ERBB2* genes, respectively (TCGA). Specifically, we analyzed the histogram of *ESR1* and *ERBB2* expression using a bin size of 0.5 (log2 unit). For both genes, we found bi-modal distributions. The thresholds were determined as the median bin value between the two peaks. We then isolated ER- tumors from both datasets, and used ERBB2 status as phenotypical labels and genes that were upregulated in MMTV-*Wnt1* mammary tumor models [29] as the gene set. The GSEA program was downloaded from Broad Institute and performed using the default setting. p values were determined empirically by random shuffling of phenotypic labels.

We obtained Level-3 (normalized) TCGA breast tumor profiles of DNA copy number, RNA, and protein expression (RPPA). ER-negative tumors were selected based on the protein level of ER determined by RPPA. Correlation between ERBB2 protein level and WNT suppressors within ER- tumors was gauged by Pearson correlation coefficients, and the corresponding p values were computed based on Student’s t tests for correlation coefficients. We also determined thresholds to classify tumors into discrete categories (e.g., ERBB2-high vs. ERBB2-low). These thresholds were defined by midlines between the two models of bi-modal distributions.

## Results

### Generation of TOP-*tva* Transgenic Mouse Lines

**Figure 1:**
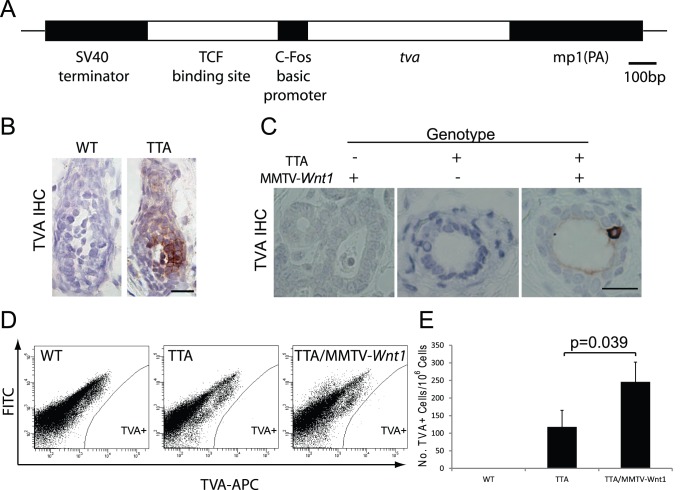
Generation of TOP-*tva* transgenic mice. (A) Diagram of the TOP-*tva* construct. (B) Immunohistochemistry staining for TVA in hair follicles of 4-day-old wild type (WT) and TOP-*tva* littermates. (C) Immunohistochemical staining for TVA in mammary glands from adult MMTV-*Wnt1*, TTA, and TTA/MMTV-*Wnt1* bi-transgenic mice. The genotypes of the samples are shown at the top. Scale bar = 20 µm. (D) Flow cytometry analysis for TVA+ cells in mammary single-cell preparations from mice of the indicated genotype. The FITC channel was used to separate autofluorescence signal. (E) Bar-graph shows quantification of TVA+ cells.

We have previously reported the use of a retrovirus method for expressing an oncogene in a specific subset of mammary gland cells in vivo [27]. This method uses a modified avian leukovirus vector (RCAS) to infect mammalian cells that are made susceptible to infection by transgenic expression of the gene encoding the RCAS receptor, TVA. To deliver oncogenes selectively into Wnt signaling-active mammary gland cells, we made a transgenic construct that expresses *tva* under the control of the TOP promoter (Figure 1A). The TOP promoter contains the *cFos* minimal promoter and a concatemer of three TCF binding motifs [31], [32]. It is the most commonly used promoter for reporting Wnt signaling in cultured cells, and has been used in transgenic animals to indicate Wnt activities in a variety of tissues [25], [33]. Pronucleus injection of the TOP-*tva* construct (TT) resulted in six potential founders that transmitted the transgene in Mendelian ratios. Using immunohistochemical staining for TVA, we found that two of them (TTA and TTB) produced TVA in precursor cells in the hair follicles (Figure 1B and Figure S1A), a site both known to have strong Wnt signaling and to produce β-gal in mice expressing the *lacZ* gene from the TOP promoter [33]. Using flow cytometry, we detected TVA in 118±47 and 181±143 cells per 10^6^ mammary epithelial cells in TTA and TTB mice, respectively (age = 10 weeks; n = 3) (Figure 1D & E; Figure S1C). These data suggest that TVA is produced in a small number of mammary epithelial cells in both TTA and TTB lines. However, by immunohistochemistry of representative sections, we did not detect TVA in either TTA or TTB mammary glands (Figure 1C; Figure S1B), not entirely surprisingly considering the rarity of this population of TVA+ cells. This is consistent with the infrequent detection of β-gal+ mammary cells in the TOPGAL model, based on experiments in our own laboratory and as reported [34].

To ascertain that the *tva* expression from this TOP-*tva* transgene is responsive to Wnt signaling, we bred TTA and TTB with MMTV-*Wnt1* transgenic mice to obtain bi-transgenic mice (age = 10 weeks; n = 3). By FACS, 2.1- and 3.9-fold more TVA+ cells per 10^6^ mammary cells were detected in TTA/MMTV-*Wnt1* and TTB/MMTV-*Wnt1* bi-transgenic mice, respectively (Figure 1D & E; Figure S1C), compared to the corresponding *tva* transgenic mice that did not carry the *Wnt1* transgene. These data indicate that these TVA+ cells are indeed responsive to Wnt stimulation. In accord, even by immunohistochemical staining, TVA+ cells could also be occasionally detected in the luminal epithelium in both bi-transgenic lines (Figure 1C & Figure S1B). Of note, the majority of mammary cells still did not produce TVA despite constitutive expression of the transgenic *Wnt1*. This is probably because only a small subset of mammary cells is capable of responding to Wnt and activating canonical Wnt signaling.

### TVA+ Mammary Cells in TOP-*tva* mice are Susceptible to RCAS Infection and can be Induced to Form Tumors by PyMT

To confirm that the TVA+ cells in these TT mice are indeed susceptible to RCAS infection and are thus suitable for RCAS-mediated genetic manipulation, six TTA mice (age = 7–10 weeks) were intraductally injected with RCAS-*GFP* (10^7^ IUs in 10 µl per gland). The injected mammary glands were collected 2.5 days later for flow cytometry analysis, and non-injected and injected wild type mouse glands were used as the reference for the negative control. Approximately 9 GFP+ cells were detected per 10^6^ mammary cells from TTA mice (Figure S2). These data demonstrate that TVA+ cells in this TTA model are susceptible to RCAS infection and can be used to mediate gene transfer by the RCAS vector. Using the same methods, we found that TTB was also susceptible to infection (Figure S3).

**Figure 2:**
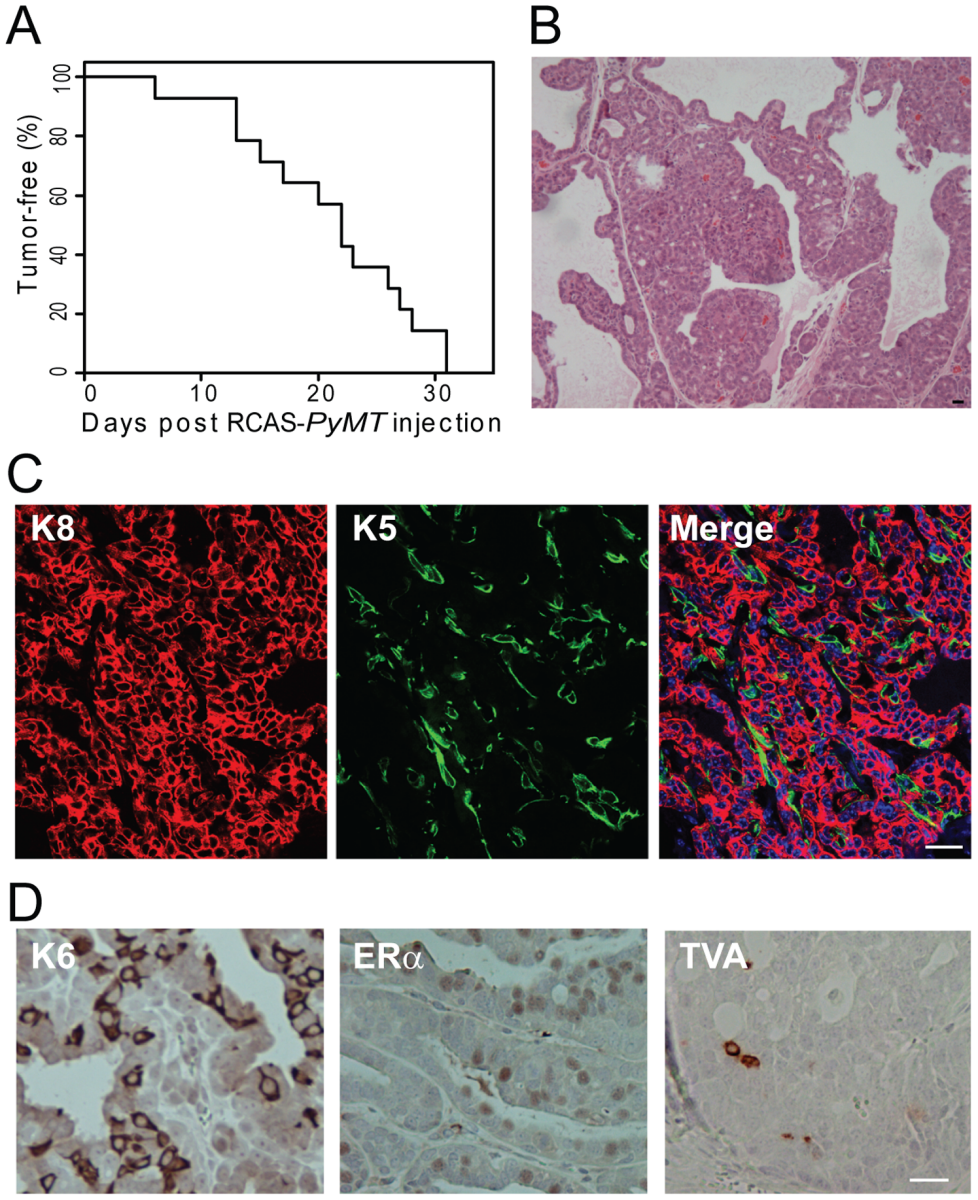
TVA+ cells in TTA mammary glands can be induced to form tumors by RCAS-*PyMT*. (A) Kaplan-Meier tumor-free survival curve of TTA mice infected with RCAS-*PyMT*. Age: 12∼16 weeks. N = 14. (B) RCAS-*PyMT*-induced tumors are adenocarcinoma. H&E staining of a representative RCAS-*PyMT*-induced tumor from TTA mice is shown. (C) Immunofluorescent staining for K8 and K5 in RCAS-*PyMT*-induced tumors. (D) Immunohistochemistry staining for K6, ERα, and TVA in RCAS-*PyMT*-induced tumors. Scale bar = 20 µm.

To validate that these TTA and TTB mice indeed express TVA and are suitable for tumor induction by RCAS-mediated expression of an oncogene, we intraductally injected them with RCAS virus carrying the gene encoding the polyoma middle T antigen (PyMT) [35]. PyMT is a viral oncoprotein that activates Src and PI3K [36], and is apparently sufficient in transforming mammary cells to cancer, when its gene is either expressed as a transgene [37] or delivered by RCAS into the mammary epithelium of MMTV-*tva* [27] or keratin 6a-*tva* transgenic lines [26]. Fourteen TTA (age = 12–16 weeks) were infected by RCAS-*PyMT* (10^7^ IUs per gland; three mammary glands per mouse). All 14 infected mice developed palpable tumors within one month with a median latency of 22 days (Figure 2A), in contrast to no tumor detection in non-transgenic mice injected with any RCAS virus including RCAS-*PyMT* ([38], [39] and data not shown). This short tumor latency in TTA mice is similar to the latency in MMTV-*tva* or keratin 6a-*tva* mice infected by RCAS-*PyMT*, confirming our previous reports that RCAS-mediated delivery of *PyMT* is sufficient to cause malignant transformation of mammary cells [26], [27]. These observations demonstrate that these *tva*-expressing Wnt-responsive cells can be induced by a potent oncogene to rapidly form mammary tumors, and that this line is suitable for RCAS-mediated oncogene expression and tumor modeling. Of note, tumors induced by RCAS-*PyMT* in TTA as well as TTB lines are papillary adenocarcinomas (Figure 2B), harboring a heterogeneous population of cells including keratin 8+ epithelial cells and keratin 5+ myoepithelial cells (Figure 2C), as well as cells stained positive for estrogen receptor α or keratin 6, a marker for biopotential mammary progenitor cells [26]. TVA+ cells were only occasionally observed (Figure 2D), indicating that the overwhelming majority of the progeny of originally infected TVA+ cells had turned into canonical Wnt pathway-inactive cells and lost *tva* expression. The histopathology and cellular heterogeneity of these tumors are very similar to the RCAS-*PyMT*-induced tumors in keratin 6a-*tva* mice [26], perhaps reflecting their similar origin in cells that are not yet differentiated.

### TVA+ Mammary Cells in TOP-*tva* mice do not Evolve into Tumors after ErbB2 Activation, While TVA+ Mammary Cells in MMTV-*tva* mice do

**Figure 3:**
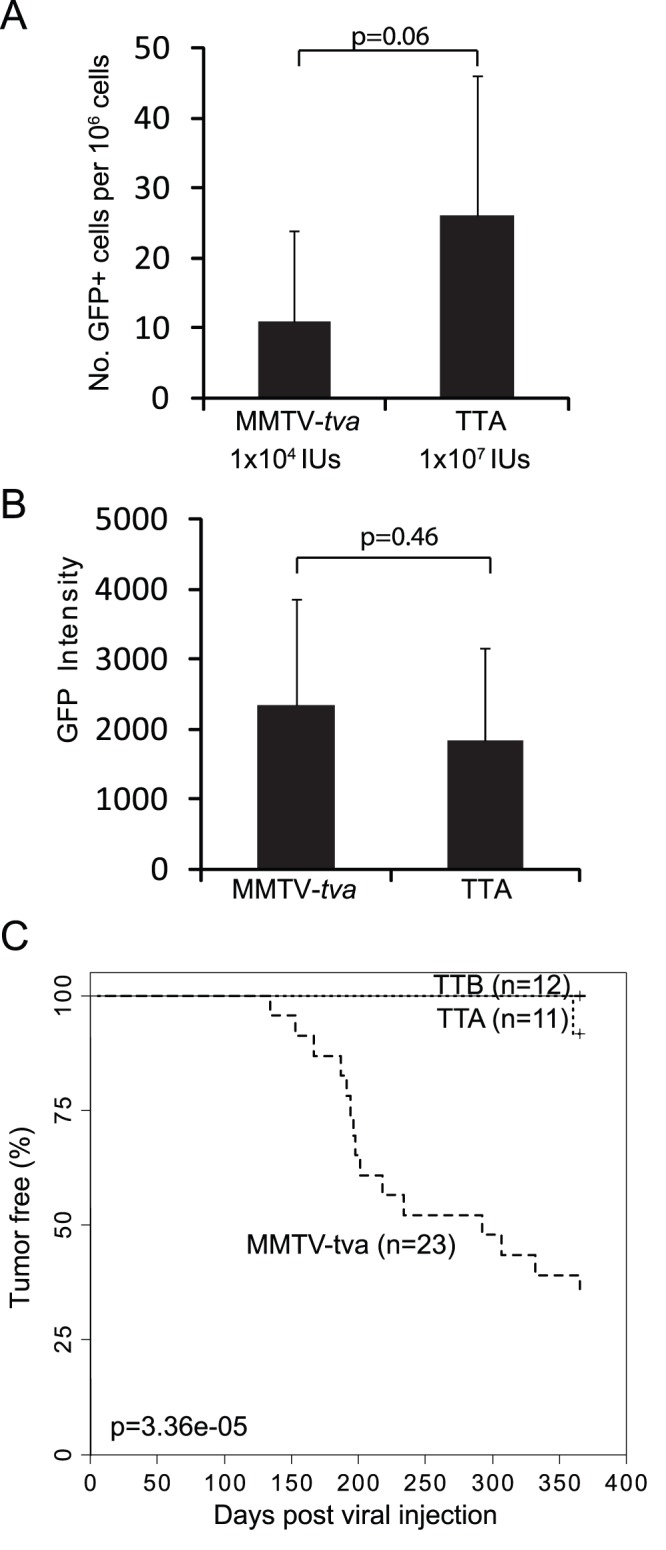
TVA+ mammary cells in TOP-*tva* mice are resistant to tumor induction by caErbB2. (A) 10^7^ and 10^4^ IUs of RCAS-*GFP* infected similar numbers of mammary gland cells in TTA and MMTV-*tva*, respectively. Of note, comparing to non-infected mammary glands, injection of 10^7 ^IUs of RCAS-GFP into non-transgenic mice did not lead to any detectable signal, indicating that the GFP signaling in this graph is specific. (B) The GFP signal intensity in RCAS-*GFP-*infected cells from TTA was compared with that from MMTV-*tva*. (C) Kaplan-Meier tumor-free survival curves of RCAS-*caErbB2*-infected mice of the indicated genotypes.

We tested whether the TVA-marked Wnt pathway-activated cells are at increased risk of transformation by an oncogene compared to other mammary epithelial cells. We have reported that RCAS carrying an activated version of *ErbB2* (RCAS-*caErbB2*) induces mammary tumors with a median latency of 6 months in MMTV-*tva* mice that express *tva* from the MMTV promoter, which is active in the great majority of cells in the mammary epithelium [27]. Therefore, we sought to determine whether RCAS- *caErbB2* may induce tumors more rapidly in TT mice than in MMTV-*tva* mice. For this comparison to be valid, the infection rates have to be similar between TT mice and MMTV-*tva* mice. TT mammary glands harbor significantly fewer TVA+ cells than the MMTV-*tva* glands, so a lower viral dosage had to be injected into MMTV-*tva* glands. In the end, we found that injecting 1×10^7^ IUs per gland in TTA or TTB (age = 12–13 weeks) reached at least the same rate of infection as injecting 1×10^4^ IUs per gland in age-matched MMTV-*tva* mice (p = 0.06 for the comparison between TTA and MMTV-*tva*; p = 0.26 for the comparison between TTB and MMTV-*tva*) (Figure 3A and Figure S3A). Furthermore, we confirmed that the average RCAS LTR promoter signal strength was similar in infected cells in TTA and TTB vs. MMTV-*tva* mice (Figure 3B and Figure S3B), which is expected from the generally ubiquitous nature of the activity of the RCAS LTR [39].

Having established the adjusted viral dosages for achieving similar rates of infected cells in these different TVA lines, we injected RCAS-*caErbB2* into MMTV-*tva* mice (n = 30; age = 12–16 weeks; one set of #2–4 glands; 1×10^4^ IUs per gland) and age-matched TTA (n = 11) and TTB (n = 12) mice (one set of #2–4 glands; 1×10^7^ IU per gland). 60% of the infected MMTV-*tva* mice developed tumors within one year; however, only one tumor was observed in the TTA group, and none was detected in the TTB groups (p<0.001 for both comparisons) (Figure 3C), despite a slightly larger population of the initially infected cells in TTA and TTB lines than in the MMTV-*tva* line. In addition, no tumor was detected in 10 additional TTA mice that were infected with the high dose RCAS-*caErbB2* (1×10^7^ IU per gland) at the age of 24–28 weeks. Collectively, these data demonstrate that the Wnt signaling-active subset of mammary cells does not evolve into tumor following activation of ErbB2.

In response to an oncogenic insult, normal cells may rapidly activate apoptosis, thus erecting a “barrier” to carcinogenesis [40], [41]. In MMTV-*tva* mice, we have reported potent apoptosis induction in some of the cells in mammary early lesions initiated by RCAS-*caErB2* [42]. Perhaps in TOP-tva mice, these TVA+ cells more strongly activate this apoptosis anticancer barrier than other mammary cells do. While it was very difficult to identify the few initially infected cells and measure their rate of apoptosis, we asked whether the few initially infected cells expanded and evolved into detectable early lesions. At necropsy of these infected TTA mice (one year after infection), we used immunohistochemical staining to search for caErbB2-positive cells and early lesions in all three infected mammary glands from each of the 8 infected mice. We did not detect any infected cells in any of these infected glands (5 sections from each gland, which are 30 µm apart). However, multiple foci of early lesions (less than 3 layers of epithelial cells) or more advanced early lesions (more than 3 layers of epithelial cells) were detected in 4 of the 8 infected MMTV-*tva* mice that failed to develop tumors (Figure S4). These data suggest that following ErbB2 activation, Wnt signaling-active mammary cells either died or failed to expand into detectable early lesions.

**Figure 4:**
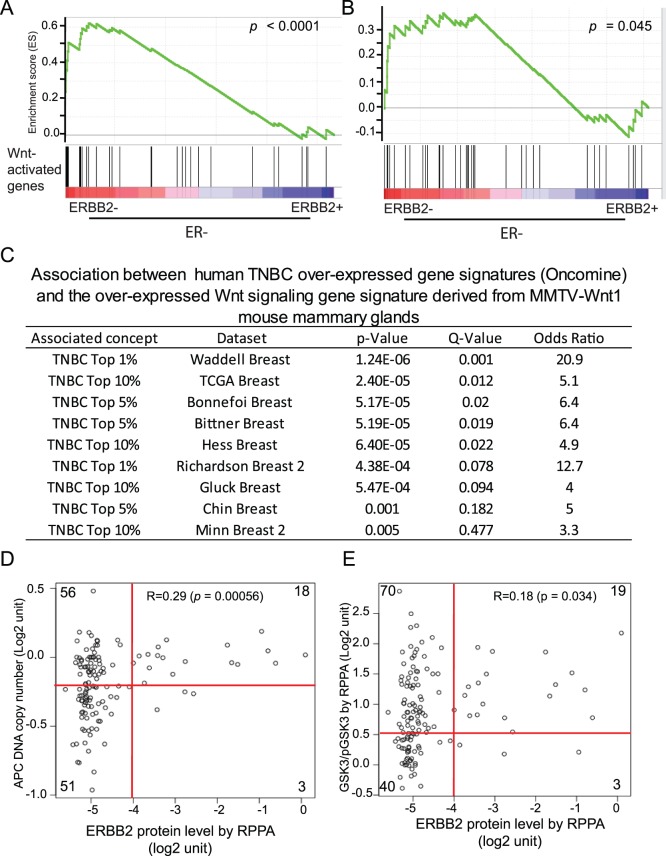
ERBB2-positive breast cancers lack evidence of the Wnt pathway activation. (A and B) Gene Set Enrichment Analysis (GSEA) for the distribution of Wnt-activated genes upregulated in ERBB2-negative vs. ERBB2-positive tumors in EMC-MSK (A) and TCGA (B) datasets. Only ER-negative tumors are included to minimize the impact of ER. The Wnt-activated gene list was obtained from our previous study [29]. The corresponding p values of the enrichment scores are shown above the plots. (C) Wnt-activated genes are associated with triple-negative, but not ERBB2-positive, breast cancers. Breast cancer concepts significantly associated with the Wnt-activated genes were generated using the Oncomine. (D and E) Scatter plots showing the correlation between ERBB2 protein levels (determined by RPPA) and two major WNT suppressors in ER-negative tumors. (D) Correlation between the APC DNA copy number and ERBB2. (E) Correlation between the functional GSK3 index (defined as GSK3/pGSK3 by RPPA) and ERBB2. Pearson correlation coefficients and the corresponding p values are shown. To illustrate the lack of *APC* DNA amplification and the low level of functional GSK3 index in ERBB2+ tumors, the plots are also divided into four quadrants with the number of events in each quadrant shown. Fisher’s exact tests were performed to test the statistical significance.

Having found that in the mouse, ErbB2 induces tumors from mammary cells other than Wnt signaling-active cells, we asked whether this cell subset preference in ErbB2-initiated tumorigenesis might also be true in human patients. First, we performed Gene Set Enrichment Analysis (GSEA) to test whether Wnt-activated genes are enriched in ERBB2-negative cases over ERBB2-positive cases among all ER-negative tumors in our previously reported expression dataset [30]. We have previously identified 58 overexpressed genes (cut-off: >1.8-fold) in mammary glands of MMTV-*Wnt1* mice compared to age-matched non-transgenic mammary glands [29]. We found that this group of Wnt-activated genes was enriched in ERBB2-negative cases, but not ERBB2-postive cases (p<0.0001). Using a similar method, we found that these Wnt-activated genes were also enriched in ERBB2-negative cases–but not ERBB2-positive cases–of the ER-negative subset of tumors in the larger TCGA dataset (cancergenome.nih.gov) (p = 0.045; Figure 4A & B). Furthermore, using this list of genes to identify associated gene signatures in Oncomine breast cancer datasets, we found that this Wnt signature did not associate with the ERBB2 subset of breast cancers, but associated strongly with the triple-negative breast cancers (Figure 4C), consistent with the previous finding that basal-like human breast cancers usually exhibited evidence of activated Wnt signaling [43], [44]. We looked at APC, which is known to suppress Wnt signaling, and found that human tumors with higher levels of ERBB2 protein (measured by RPPA) did not show the copy number loss of *APC* frequent in tumors with lower ERBB2 (p = 0.00056) (Figure 4D), and that higher ERBB2 was associated with higher *APC* expression (R = 0.25; p = 0.0015) (data not shown). Higher ERBB2 protein levels were also correlated with higher levels of unphosphorylated GSKβ (R = 0.18; p = 0.034), which targets β-catenin for degradation and thus inactivates Wnt signaling (Figure 4E). Taken together, these data strongly suggest that in human breast tumors, activation of ERBB2 is not associated with activation of Wnt signaling; thus, ERBB2-positive breast cancers in human, as in mice, may have an origin in non-Wnt-activated breast cells.

## Discussion

We and others have reported that after transgenic or virus-mediated activation of ErbB2 in the general mammary epithelium, mammary tumors rapidly develop [27], [45], [46]. However, the in vivo experiments presented in this report identified a subset of mammary cells that failed to evolve into tumors following ErbB2 activation. These results provide direct in vivo evidence that mammary epithelial cells are not equal in their response to oncogene-initiated transformation. These data also suggest that different subsets of breast cancers may have distinct cells of origin, as previously suggested by us and others [47], [48]. Although our experiments using the MMTV-*tva* line did not directly identify the type of mammary cells that eventually evolved into a tumor, the comparison of tumor latency between MMTV-*tva* and TOP-*tva* mice strongly implies that the cell of origin in RCAS-*caErbB2*-infected mice was Wnt signaling-inactive cells.

Our data also suggest that the ERBB2+ subset of human breast cancer may arise from breast cells that are low in Wnt signaling. Indeed, this subset of human breast cancers lacked evidence of active Wnt signaling (Fig 4). In accord with our finding, it has been reported that β-catenin was excluded from forming a heterodimer with TCF/LEF in some human ErbB2+ breast cancer cell lines [49]. This previous report also casts uncertainty on the significance of the reported accumulation of some components of the Wnt pathway in human breast cancers [50], [51]. However, there are some reports suggesting that β-catenin activity may play a role in ErbB2+ mammary tumors [50], [52]–[54], while multiple other studies suggest that the Wnt signaling-active subset of breast cells may be especially vulnerable to developing into basal tumors, which exhibit increased Wnt signaling [20], [55], [56].

Our in vivo evidence for ErbB2+ tumors arising from Wnt signaling-inactive mammary epithelial cells is consistent with our previous reports and others on the potential cell origin of ErbB2-initiated mammary tumors. In examining the cellular heterogeneity of mammary tumors and production of progenitor and stem cell markers in six transgenic models of breast cancer, we found evidence that mammary tumors arising in MMTV-*ErbB2* mice may have an origin in more differentiated mammary luminal epithelial cells [55], which probably lack Wnt signaling since Wnt signaling is usually found in stem and early progenitor cells [19]. In studying MMTV-*ErbB2* mice that were crossed to mice with a WAP-*Cre* transgene and the R26R allele (which expresses *lacZ* only after an intervening floxed DNA fragment between the ROSA promoter and *lacZ* is deleted by Cre), Henry et al. [57] found β-galactosidase activity in mammary early lesions, suggesting that ErbB2 has an increased transforming potential in cells that express *WAP* (which defines a subset of relatively differentiated luminal epithelial cells in virgin mice) and in their progeny. However, the idea of a more differentiated cell of origin for mammary tumors in MMTV-*ErbB2* mice is not without controversy: a progenitor cell of origin has also been reported [58].

There may be several reasons that could explain why following ErbB2 activation, the Wnt signaling-active mammary cells fail to evolve into tumors. We have recently reported mammary anticancer barriers (apoptosis and cell cycle arrest) that are erected following ErbB2 activation [59]. Perhaps, these barriers are activated to higher levels in these Wnt signaling-active mammary cells, thus killing them and preventing them from evolving into cancer. It is also possible that in this subset of mammary cells, aberrant ErbB2 failed to potently activate downstream oncogenic signaling and therefore failed to induce cell expansion and transformation. In the intestinal epithelium, activated Ras, a crucial downstream component of the ErbB2 oncogenic signaling network, fails to activate Raf/MEK/ERK signaling and cannot transform the intestinal epithelium [60]. We do not yet know how these Wnt signaling-active cells would respond to other oncogenic events. It is also possible that forced over-activation of Wnt signaling can transform them to malignancy–the Wnt signaling-active cells in the intestinal tissue are highly susceptible to transformation by inactivation of *APC* [18]. Perhaps upon aberrant stimulation with Wnt, these TOP-tva-expressing mammary cells, as well as other mammary cell subsets, can also become primed for tumor induction by ErbB2. We have reported that in the MMTV-*Wnt1* transgene background, either MMTV-*ErbB2* or RCAS-*caErbB2* can rapidly induce mammary tumors [27], [61]. Likewise, in intestinal and renal epithelium that is null for *APC*, Ras activation causes strong activation of Raf/MEK/ERK signaling and rapid carcinogenesis [60]. Of note, TVA production in our TOP-*tva* mice may label only a subset of mammary cells that are Wnt signaling-active. It has been reported that many more cells, usually in the basal layer, express *Axin2*, a transcriptional target of Wnt signaling that is frequently used to mark Wnt signaling-active cells in several tissues [19]. It remains to be tested whether these cells are more or less susceptible to ErbB2-initiated tumorigenesis than the bulk of the mammary epithelium.

In conclusion, mammary cells are not equal in their susceptibility to tumor initiation by an oncogenic event. Unlike other mammary epithelial cells, the Wnt signaling-active mammary cells defined by TOP activity fail to evolve into tumors following aberrant activation of oncogenic ErbB2 signaling. Therefore, ErbB2+ human breast cancers may have a different cell of origin from the Wnt-signaling active, basal-like subtype of human breast cancer.

## Supporting Information

Figure S1 **Characterization of the TTB transgenic line.**
(EPS)

Figure S2 **TVA+ cells in TTA mammary glands are susceptible to RCAS virus infection.**
(EPS)

Figure S3 **Comparison of RCAS-** ***GFP*** **infection rates and intensities between TTB and MMTV-** ***tva*** **mouse mammary glands.**
(EPS)

Figure S4 **There is no detectable RCAS-ErbB2 infected cell in the mammary glands of TT mice which failed to develop tumor one year after infected by RCAS-** ***ErbB2*** **.**
(TIF)
