# Supplementary figures and images for: Mammary Cells with Active Wnt Signaling Resist ErbB2-Induced Tumorigenesis
Source: PLoS One. 2013 Nov 12;8(11):e78720. doi: 10.1371/journal.pone.0078720 (PMC3827100; doi:10.1371/journal.pone.0078720)

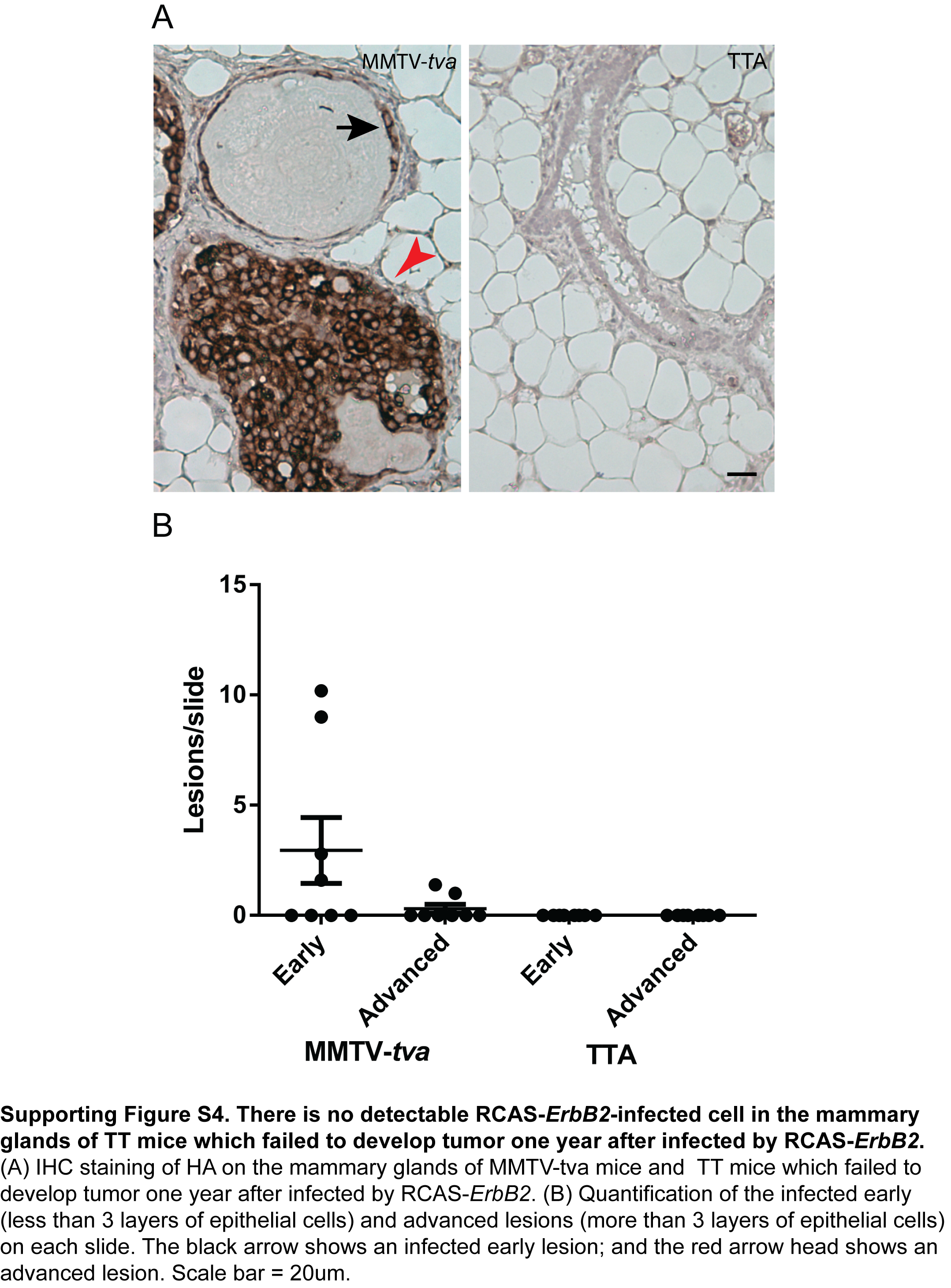

Supplement: Figure S4 — There is no detectable RCAS-ErbB2 infected cell in the mammary glands of TT mice which failed to develop tumor one year after infected by RCAS- ErbB2 . (TIF) [file pone.0078720.s004.tif]
